# Supplementary material for: Tryptophan Hydroxylase 2 Knockout Male Rats Exhibit a Strengthened Oxytocin System, Are Aggressive, and Are Less Anxious
Source: ACS Chem Neurosci. 2022 Oct 5;13(20):2974–81. doi: 10.1021/acschemneuro.2c00448 (PMC9585586; doi:10.1021/acschemneuro.2c00448)
Supplement: Supplementary file 1 — cn2c00448_si_001.pdf [file cn2c00448_si_001.pdf]

Supporting Information

TITLE:

Tryptophan Hydroxylase 2 Knockout Male Rats Exhibit a Strengthened Oxytocin System, are Aggressive, and Less Anxious

Xianzong Meng[1], Joanes Grandjean[1,3], Giulia Sbrini[2], Pieter Schipper[1], Nita Hofwijks[1], Jesse Stoop[1], Francesca Calabrese[2], Judith Homberg[1]*

Contents:

Figure S1

Figure S2

Affiliation:

1. Department of Cognitive Neuroscience, Donders Institute for Brain, Cognition, and Behaviour, Radboud University Medical Centre, 6525 AJ Nijmegen, The Netherlands.

2. Department of Pharmacological and Biomolecular Sciences, Università Degli Studi Di Milano, Via Balzaretti 9, 20133, Milan, Italy

3. Department of Medical Imaging, Radboud University Medical Centre, 6525 GA Nijmegen, The Netherlands.

*Correspondence: Judith.Homberg@radboudumc.nl

Supplementary materials

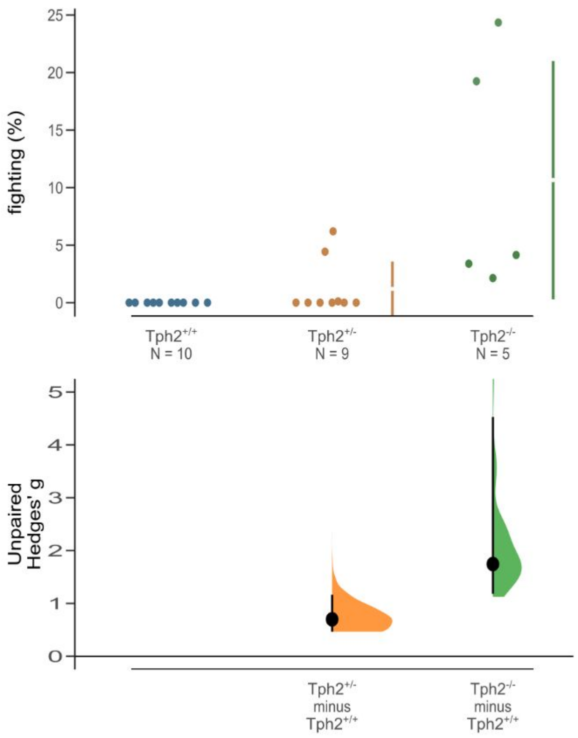

Figure S1. Fighting during social behavior test. $n_{Tph2+/+}$ = 10, $n_{Tph2-/-}$ = 7, $n_{Tph2+/-}$ = 9. The Hedges' g for 2 comparisons against the shared control Tph2$^{+/+}$ are shown in the Cumming estimation plot. The raw data are plotted on the upper axes. On the lower axes, mean differences are plotted as bootstrap sampling distributions. Each mean difference is depicted as a dot. Each 95% confidence interval is indicated by the ends of the vertical error bars.

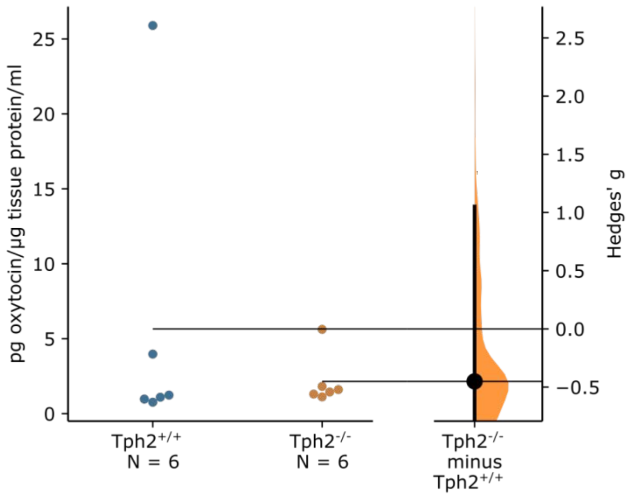

Figure S2. Oxytocin expression in dorsal raphe nucleus. n = WT (6), Tph2-/- (6). The Hedges' g between Tph2+/+ and Tph2-/- is shown in the above Gardner-Altman estimation plot. Both groups are plotted on the left axes; the mean difference is plotted on floating axes on the right as a bootstrap sampling distribution. The mean difference is depicted as a dot, the 95% confidence interval is indicated by the ends of the vertical error bar.
